# Neurons in Vulnerable Regions of the Alzheimer’s Disease Brain Display Reduced ATM Signaling^1^,^2^,^3^

**DOI:** 10.1523/ENEURO.0124-15.2016

**Published:** 2016-02-27

**Authors:** Xuting Shen, Jianmin Chen, Jiali Li, Julia Kofler, Karl Herrup

**Affiliations:** 1 Division of Life Science, Hong Kong University of Science and Technology, Clear Water Bay, Kowloon, Hong Kong, China; 2 Department of Otolaryngology, Xiangya Hospital, Central South University, Changsha 410008, Hunan, China; 3 Department of Cell Biology and Neuroscience, Rutgers University, Piscataway, New Jersey 08854; 4 Key Laboratory of Animal Models and Human Disease Mechanisms of Chinese Academy of Sciences, and Yunnan Province, Kunming Institute of Zoology, Kunming 650223, Yunnan, China; 5 Department of Pathology and The Alzheimer’s Disease Research Center, University of Pittsburgh School of Medicine, Pittsburgh, Pennsylvania 15213

**Keywords:** ataxia-telangiectasia, cell cycle, EZH2, HDAC4, neurodegeneration

## Abstract

Ataxia telangiectasia (A-T) is a multisystemic disease caused by mutations in the ATM (A-T mutated) gene. It strikes before 5 years of age and leads to dysfunctions in many tissues, including the CNS, where it leads to neurodegeneration, primarily in cerebellum. Alzheimer’s disease (AD), by contrast, is a largely sporadic neurodegenerative disorder that rarely strikes before the 7th decade of life with primary neuronal losses in hippocampus, frontal cortex, and certain subcortical nuclei. Despite these differences, we present data supporting the hypothesis that a failure of ATM signaling is involved in the neuronal death in individuals with AD. In both, partially ATM-deficient mice and AD mouse models, neurons show evidence for a loss of ATM. In human AD, three independent indices of reduced ATM function—nuclear translocation of histone deacetylase 4, trimethylation of histone H3, and the presence of cell cycle activity—appear coordinately in neurons in regions where degeneration is prevalent. These same neurons also show reduced ATM protein levels. And though they represent only a fraction of the total neurons in each affected region, their numbers significantly correlate with disease stage. This previously unknown role for the ATM kinase in AD pathogenesis suggests that the failure of ATM function may be an important contributor to the death of neurons in AD individuals.

## Significance Statement

The immediate cause of the death of neurons in Alzheimer’s disease (AD) is currently unknown. We show that, in vulnerable regions of the human AD brain, increasing numbers of neurons undergo an unexpected loss of ATM (ataxia-telangiectasia mutated) function as the disease progresses. Total ATM levels drop, and multiple lines of evidence reveal that ATM function is lost coordinately in at-risk neurons. The replication of this phenotype in the hippocampus of three different AD mouse models suggests that it is central to the neuronal death mechanism. These data offer new approaches toward understanding the mechanisms of neuronal cell loss in Alzheimer’s disease.

## Introduction

Alzheimer’s disease (AD) is the major cause of dementia in the elderly. Its prevalence and long disease course confer a significant burden on the individuals affected, their caregivers, and society. The clinical dementia of AD is associated with a progressive neurodegeneration that is characterized by pathological hallmarks, including extracellular senile plaques (β-amyloid deposits), neurofibrillary tangles (hyperphosphorylated tau), as well as synaptic and neuronal loss (Querfurth and LaFerla, 2010). In addition, in AD as well as other neurodegenerative diseases, a significant fraction of the neurons in populations at risk for death display evidence of having re-entered a cell cycle (Smith and Lippa, 1995; McShea et al., 1997; Nagy et al., 1997; Busser et al., 1998; Yang et al., 2001, 2003; Nguyen et al., 2002; Lim and Qi, 2003; Arendt, 2009). This abortive attempt to divide is believed to be lethal for adult neurons, *in vivo* and *in vitro*.

Ataxia-telangiectasia (A-T) is a rare autosomal genetic disease of childhood. The affected gene encodes a large PI3 kinase family member known as ATM (A-T mutated). ATM is a key cellular protein involved in cell cycle checkpoint control during the repair of DNA damage. Activated by double-strand DNA breaks, the ATM kinase phosphorylates a number of downstream targets involved in DNA damage repair, cell cycle arrest, and apoptosis (Shiloh and Rotman, 1996; Barlow et al., 1997; Khanna, 2000; Lavin and Kozlov, 2007). Deficiency in this DNA damage response is often cited as one reason why individuals with A-T experience a higher incidence of cancer. Yet, because of the lethal consequences of ectopic expression of cell cycle markers in neurons (Park et al., 1997; Copani et al., 2001; Yang et al., 2001; Nagy, 2005), it has also been hypothesized that compromise of this cell cycle checkpoint function has independent relevance for the neurodegeneration phenotype (Yang and Herrup, 2005). As an example, cerebellar Purkinje cells die in substantial numbers during the course of human A-T, and their deaths are associated with unscheduled cell cycle events (CCEs). Tellingly, Purkinje cell CCEs are found in genetically engineered *Atm^−/−^* mouse models as well as in human A-T. The suggestion is that the loss of ATM protein, or its function, in vulnerable populations of neurons leads to a loss of cell cycle control and, ultimately, to cell death.

Nothing about this model suggests that it applies only at childhood ages or only to one cell type, the Purkinje cell. Indeed, the evidence shows that suppression of the neuronal cell cycle is a life-long requirement for the neurons of the normal adult brain. Thus, while genetic deficiency of ATM leads to an early childhood neurodegenerative syndrome, if sporadic loss of ATM function in individual neurons were to occur later in life, the resulting ATM deficiency might be an unsuspected part of the mechanism leading to loss of neuronal cell cycle control and, ultimately, to cell death. Therefore, we tested the hypothesis that a neuronal ATM deficiency might be involved in the neurodegeneration found in Alzheimer’s disease. One challenge faced in exploring this idea is that CCEs affect only a small fraction (∼10%) of the neurons in either A-T or AD (Yang et al., 2003; Yang and Herrup, 2005, 2007). Therefore, we took advantage of recent findings that ATM regulates the levels of the histone methyltransferase EZH2 (enhancer of zeste homolog 2; Li et al., 2013), as well as the cytoplasmic location of histone deacetylase 4 (HDAC4; Li et al., 2012). This idea had been tested previously for HDAC4 (Herrup et al., 2013) and had been shown to be a practical approach. In the current study, we both validate and extend these earlier findings. We use three independent measures of ATM function, and show that in multiple brain regions affected during the course of AD a fraction of the constituent neurons shows decreased ATM protein and decreased ATM signaling. This same phenotype is found in the three separate AD mouse models. We thus propose that the loss of ATM function is a key part of the mechanism of neuronal death found in Alzheimer’s disease.

## Materials and Methods

### Human subjects

Paraffin-embedded 10 μm brain sections were from the following sources with approval from the appropriate local regulatory authorities. We examined 27 case patients graciously provided by the University of Pittsburgh Alzheimer’s Disease Research Center (ADRC) brain bank with approval from the Committee for Oversight of Research and Clinical Training Involving Decedents (CORID). Each case had been diagnosed neuropathologically and ranked by Braak stage. Nine individuals had Braak stage I–II disease [none or low tau pathology (NL)]; nine had disease in stage III–IV [moderate tau pathology (M)]; and nine had disease in stage V–VI [advanced (AD-like) tau pathology (AD)]. Basic information is shown in Table 1. Additional frozen tissue was a generous gift of the ADRC at Washington University in St. Louis (Grant P50-AG-05681) with approval from the Neuropathology Core (protocol #T1016).

**Table 1:** Braak stage grouping and age distribution of case patients enrolled in immunohistochemistry experiment

Group	Braak stages	Gender	Age (years) (mean ± SEM)
NL	I–II	8 M/1 F	78 ± 3
M	III–IV	8 M/1 F	85 ± 2
AD	V–VI	5 M/4 F	81 ± 1.5

F, Female; M, male.

### Animals

All animals were housed in the accredited animal facility at universities of the authors. All procedures involving animals were approved by the respective local committees following the guidelines from local authorities. In the writing of the article, every effort has been made to follow the ARRIVE guidelines (http://www.nc3rs.org.uk/arrive-guidelines).

#### Alzheimer transgenic mice

The following three AD mouse models were used: R1.40, B6.129-Tg(APPSw)40Btla/J, C57BL/6J; PS/APP, B6.Cg-Tg(APPswe,PSEN1dE9)85Dbo/Mmjax C57BL/6J; and 3xTg, B6;129-PSEN1tm1MpmTg(APPSwe,taulP301L)1Lfa/Mmjax.

Colonies were obtained from The Jackson Laboratory. Animals of either sex were killed at 12-14 months of age. C57BL/6J mice were used as age-matched controls.

#### *Atm*-deficient mice

A breeding colony of mice with a targeted disruption of the *Atm* gene (Barlow et al., 1996) was obtained originally from The Jackson Laboratory (129S6/SvEvTac-*Atm^tm1Awb^/J*). The colony was maintained by intercrossing heterozygous *Atm^+/−^* males and *Atm^+/−^* females. Genotyping was performed on extracted tail DNA using PCR techniques described previously (Barlow et al., 1996). For this experiment, animals of either sex were killed at 2-3 months of age along with age-matched controls.

### Primary cortical neuronal culture

Embryonic cortical neurons were isolated by standard procedures. Embryonic day 16.5 (E16.5) embryonic cerebral cortices were treated with 0.25% trypsin-EDTA (ThermoFisher Scientific) and dissociated into single cells by gentle trituration. Cells were suspended in Neurobasal medium supplemented with B27 and 2 mm GlutaMAX (ThermoFisher Scientific), then they were plated on coverslips or dishes coated with poly-l-lysine (0.05 mg/mL; ThermoFisher Scientific). All cultures were grown for a minimum of 14 d *in vitro* before harvest. Genotyping was performed after plating the neurons.

### Immunohistochemistry: paraffin

Sections were deparaffinized in xylene and rehydrated through graded ethanols (100%, 95%, 70%, and 50% to water). After antigen retrieval with citrate buffer, pH 6.0 (for 15 min at 95°C), endogenous peroxidase was quenched with 3% hydrogen peroxide for 10 min at room temperature. Sections were then blocked by 10% serum, appropriate to the antibody used, and diluted in PBS with 0.3% Triton X-100. After 1 h, sections were incubated in primary antibody diluted in blocking solution at 4°C overnight. The following day, horseradish peroxidase (HRP)-linked secondary antibody and ABC reagent (catalog #PK-6100 or #PK-6102, Vector Laboratories) were prepared according to the manufacturer instructions. After 1 h of incubation with secondary antibody, slides were immersed in ABC solution for 1 h at room temperature and visualized by DAB (catalog #SK-4100, Vector Laboratories) or VIP (catalog #SK-4600, Vector Laboratories). After counterstaining with hematoxylin (catalog # S3309, Dako), slides were dehydrated in a series of ethanols, cleared in xylene, and mounted with permanent mounting medium (catalog #H-5000, Vector Laboratories).

For double labeling, TBS was used as a wash buffer. After visualization of the first antigen with DAB, samples were blocked for a second time, incubated in the second primary antibody at 4°C overnight followed by incubation with secondary antibody and ABC reagent. Vector blue (catalog #SK-5300, Vector Laboratories) or VIP was used for visualization. No counterstain was used on sections visualized by Vector blue. Negative controls were stained by the same procedure but without primary antibody. For α-synuclein detection, sections were immunostained with LB509 following a 5 min pretreatment with protease XXIV (Sigma-Aldrich). The severity of α-synuclein pathology was scored semi-quantitatively following consensus guidelines (McKeith et al., 2005).

### Tissue histology and immunohistochemistry: frozen sections

Anesthetized mice were perfused with cold PBS, followed by 4% paraformaldehyde (PFA) in 0.1 m PBS. After perfusion, the brains were dissected, immersed in 4% PFA at 4°C overnight, then cryoprotected in 30% sucrose at 4°C overnight, followed by embedding in OCT. After sectioning at 10 µm and air drying, samples were used immediately for immunohistochemistry or stored at −80°C. For immunolabeling, after rinsing in PBS, sections were immersed in prewarmed citrate buffer at 95°C for 10 min. Protein blocking was performed before overnight simultaneous incubation with all primary antibodies. Fluorescent secondary antibodies were added for 2 h at room temperature before rinsing and counterstaining with DAPI. Sections were mounted with antifading fluorescence medium (catalog #H-1000, Vector Laboratories).

### 5-Ethynyl-2'-deoxyuridine proliferation assay

Ten micromolar 5-ethynyl-2'-deoxyuridine (EdU) was added to the cell culture for 24 h for incorporation into the genome of cells undergoing DNA replication. EdU labeling was performed according to the manufacturer protocol of the Click-iT EdU Cell Proliferation Assay Kit (catalog #C10340, ThermoFisher Scientific) as a tool for monitoring cell cycle re-entry in cortical neurons. After EdU labeling, the samples were processed for immunofluorescence or DAPI labeling before mounting.

### Immunofluorescence

Immunofluorescence was performed according to standard methods. Cells were blocked in 5% donkey serum diluted in PBS containing 0.3% Triton X-100 for 1 h at room temperature and incubated with primary antibodies overnight. After rinsing in PBS, they were incubated for 1 h at room temperature with secondary antibodies. Cells were then rinsed in PBS and counterstained with DAPI for 3 min at room temperature. After rinsing, all coverslips were mounted with antifading hard-set fluorescence medium (catalog #H-1400, Vector Laboratories) on glass slides.

### Antibodies for histological studies

The following primary antibodies were used: proliferating cell nuclear antigen (PCNA) was purchased from Cell Signaling Technology; cyclin A was purchased from Santa Cruz Biotechnology; HDAC4, ATM2C1, Ki67, cyclin A2, H3K27me3, and MAP2 were purchased from Abcam; and LB509 and AT8 were purchased from ThermoFisher Scientific. PHF1 was a generous gift from Dr. Peter Davies (Albert Einstein College of Medicine, Bronx, NY).

Secondary antisera conjugated with fluorescent Alexa Fluor dyes 488 and 647, and Cy3 were purchased from ThermoFisher Scientific and Jackson ImmunoResearch.

### Data collection and analysis

Stained human sections were photographed on an Olympus DP80 camera at a final magnification of 200× or 400×. For single-antigen labeling, only cells whose pattern of hematoxylin staining identified them as neurons were counted. Within the hippocampus, we identified the CA2 subfield according to the arrangement and morphology of neurons. In each section analyzed, all neurons located in the defined CA2 subfield were counted. For frontal cortex, we defined layer III (L3) or layer V by the size and positioning of the neurons; in each layer, we chose 16 randomly distributed fields for counting. In each section through the locus ceruleus (LC), all large neurons (identified by both hematoxylin and cytoplasmic neuromelanin) were counted. In sections through the cerebellar cortex, Purkinje cells were identified within four to five separate folia. For each folium, we randomly chose four fields for analysis.

For stained mouse brain sections, both in frontal cortex and hippocampus, total MAP2-labeled neurons within layers II to V that colocalized with markers of interest were counted at 20×. For neuronal cultures, five fields were randomly chosen for quantification using a 20× objective on an Olympus fluorescent microscope, and the percentage of the positive cells with markers of interest were counted and expressed as a fraction of the total number of MAP2-labeled neurons.

An unpaired two-tailed Student’s *t* test (Prism, version 5, GraphPad Software) was applied to determine the differences between different groups. Samples where the *p* value was <0.05 were considered statistically significant.

### Western blots

Frozen frontal cortex and cerebellar tissues were from the Washington University in St. Louis Alzheimer’s Disease Research Center. Frozen tissues were weighed and homogenized (1:10, w/w) in RIPA lysis buffer (ThermoFisher Scientific) with protease and phosphatase inhibitors (Roche). Lysates were then sonicated briefly on ice and centrifuged at 4°C, and supernatants were collected. Samples were diluted with 2× Laemmli Sample Buffer (Bio-Rad), then denatured at 95°C. Proteins were separated on 6% acrylamide SDS denatured gel and transferred to a nitrocellulose membrane (Bio-Rad). Membranes were blocked with TBST containing 5% milk (Bio-Rad) and then incubated with 1 µg/ml ATM2C1 antibody (Abcam) in blocking buffer at 4°C overnight. After 1 h of incubation in HRP-conjugated secondary antibodies, protein levels were visualized using a SuperSignal West Femto kit (ThermoFisher Scientific). The intensities of the bands were quantified using ImageJ and were normalized to actin.

### RT-PCR

Reverse transcription reactions were performed according to standard procedures. Total RNA from control and AD frozen frontal cortex was prepared using the PureLink micro-to-midi total RNA purification system (ThermoFisher Scientific). RT-PCR was performed with the Superscript III one-step RT-PCR system with platinum Taq High Fidelity (ThermoFisher Scientific). For human samples, the mRNA level of tubulin was used as an internal control. The sequences of the primers used were as follows: for *Atm* exons 14-15, 5'-ttacaaattcagaaactcttg-3' (sense) and 5'-cttggtacagttgctcaagca-3' (antisense); for *Atm* exons 34-41, 5'-aggctgttggaagctgcttg-3' (sense) and 5'-ctagtaatgggttgtaacatc-3' (antisense); for *Atm* exons 55-58, 5'-gtggaccacacaggagaatat-3' (sense) and 5'-aatagaagaagtagctacact-3' (antisense); and for *tubulin*, 5'-tggagccgggaataactg-3' (sense) and 5'-gcctcgtcctcgccctcctc-3' (antisense).

The RT-PCR reaction program was as follows: 55°C for 30 min and 94°C for 3 min, followed by 35 cycles of 94°C for 30 s, 55°C for 30 s, and 68°C for 1-2 min with an extension at 68°C for 10 min. The PCR products were analyzed on 1.5% agarose gels stained with ethidium bromide.

## Results

### Partial ATM deficiency drives neuronal cell cycle reentry and epigenetic change

**Figure 1.**
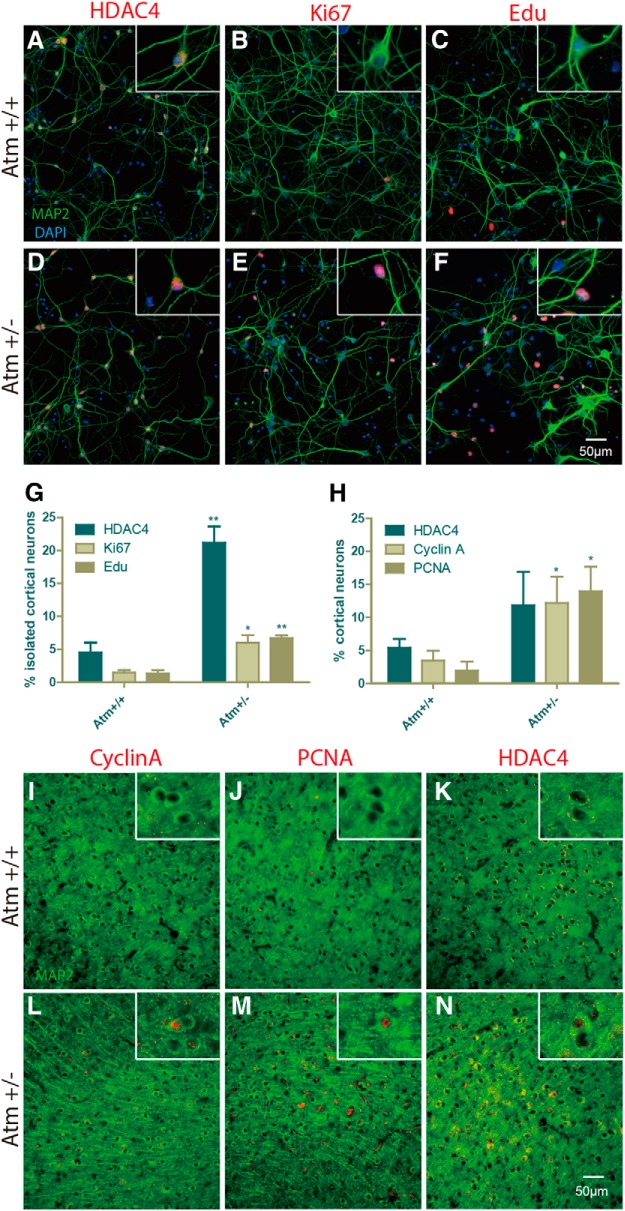
Partial ATM deficiency is sufficient to induce neuronal cell cycling and nuclear HDAC4 translocation in frontal cortex. Dissociated neurons from E16.5 mouse cortex show the impact of the partial genetic reduction in ATM. ***A–C***, Cultures of wild-type neurons show low levels of HDAC4_N_ (***A***), Ki67 (***B***), or EdU incorporation (***C***). ***D–F***, Parallel cultures of *Atm^+/−^* neurons, by contrast, have enhanced HDAC4_N_ (***D***), Ki67 (***E***), and EdU incorporation (***F***). ***G***, Quantification of the results in ***A–F***. Immunostained sections of *Atm^+/−^* mouse cortex show a similar pattern. ***I–K***, Sections of wild-type cortex show little evidence for cyclin A (***I***), PCNA (***J***), or HDAC4_N_ (***K***) immunostaining. ***L–N***, Immunostained sections of *Atm* ^+/−^ cortex, by contrast, have enhanced cyclin A (***L***), PCNA (***M***), and HDAC4_N_ (***N***) immunostaining. ***H***, Quantification of the results in ***I–K***. Scale bar, 50 µm. Difference determined by *t* test, *p < 0.05, **p < 0.01 (*n* = 3-4).

Neuronal cell cycle reentry and HDAC4 nuclear translocation are two cellular events found in both A-T patients and *Atm^−/−^* mouse models (Yang and Herrup, 2005; Li et al., 2011a, 2013; Yang et al., 2014; Hui and Herrup, 2015). Further, in both humans and mice, heterozygote carriers display some symptoms of ATM deficiency. For example, carrier human lymphocytes show increased lymphocyte radiosensitivity (Ideno et al., 2012; Godyń et al., 2016), and in heterozygous *Atm^+/−^* mice, cerebellar Purkinje cells show a loss of cell cycle control (Yang and Herrup, 2005). If our hypothesized link between neuronal ATM deficiency and neurodegeneration in AD is correct, then partial ATM deficiency might be sufficient to induce some amount of neuronal damage. We tested this idea both in cultures of dissociated mouse cortical neurons as well as in cryostat sections from heterozygous *Atm^+/−^* mouse brain. *In vitro*, both HDAC4 nuclear translocation and cell cycle re-entry (assessed by Ki67 immunostaining and EdU incorporation) were increased in *Atm^+/−^* neurons (Fig. 1*A–G*, Table 2). Similar results were obtained *in vivo*, where two cell cycle markers, cyclin A and PCNA, were both significantly elevated in *Atm^+/−^* cortex. HDAC4 nuclear translocation was also increased, although the results were not statistically significant (Fig. 1*H–N*). Thus, by these measures, even partial ATM deficiency is enough to significantly increase neuronal vulnerability. It should be noted that the reduction in ATM activity in these heterozygous *Atm^+/tm1Awb^* mice is predicted to be modest and certainly less than the 50% reduction that would be expected with a true null allele. This is because *Atm^tm1Awb^* is a hypomorphic allele that leads to reduced levels of a truncated ATM protein that retains at least some kinase activity (Li et al., 2011a). The implication is that even modest reductions in neuronal ATM function are enough to induce the phenotypes we use as markers for the loss of ATM activity.

**Table 2:** Statistical table

	Data structure	Type of test	Power
a (Fig. 1G)	Normally distributed	Unpaired *t* test	1.00
b (Fig. 1G)	Normally distributed	Unpaired *t* test	0.97
c (Fig. 1G)	Normally distributed	Unpaired *t* test	1.00
d (Fig. 1H)	Normally distributed	Unpaired *t* test	0.70
e (Fig. 1H)	Normally distributed	Unpaired *t* test	0.96
f (Fig. 2E)	Normally distributed	Unpaired *t* test	0.36
g (Fig. 2E)	Normally distributed	Unpaired *t* test	1.00
h (Fig. 2E)	Normally distributed	Unpaired *t* test	0.93
i (Fig. 2E)	Normally distributed	Unpaired *t* test	0.49
j (Fig. 2E)	Normally distributed	Unpaired *t* test	0.60
k (Fig. 3G)	Normally distributed	Unpaired *t* test	1.00
l (Fig. 3G)	Normally distributed	Unpaired *t* test	1.00
m (Fig. 3G)	Normally distributed	Unpaired *t* test	0.87
n (Fig. 3H)	Normally distributed	Unpaired *t* test	1.00
o (Fig. 3H)	Normally distributed	Unpaired *t* test	0.90
p (Fig. 4G)	Normally distributed	Unpaired *t* test	0.68
q (Fig. 4G)	Normally distributed	Unpaired *t* test	0.56
r (Fig. 4G)	Normally distributed	Unpaired *t* test	1.00
s (Fig. 4H)	Normally distributed	Unpaired *t* test	0.99
t (Fig. 4H)	Normally distributed	Unpaired *t* test	0.64
u (Fig. 4H)	Normally distributed	Unpaired *t* test	0.89
v (Fig. 4J)	Normally distributed	Unpaired *t* test	0.80
w (Fig. 4K)	Normally distributed	Unpaired *t* test	0.93
x (Fig. 4K)	Normally distributed	Unpaired *t* test	0.89
y (Fig. 4K)	Normally distributed	Unpaired *t* test	0.91
z (Fig. 4K)	Normally distributed	Unpaired *t* test	0.95
aa (Fig. 5A)	Normally distributed	Unpaired *t* test	0.52
ab (Fig. 5A)	Normally distributed	Unpaired *t* test	1.00
ac (Fig. 6D)	Normally distributed	Unpaired *t* test	0.76
ad (Fig. 7D)	Normally distributed	Unpaired *t* test	0.97
ae (Fig. 7D)	Normally distributed	Unpaired *t* test	0.84

### Neuronal ATM is reduced in mouse models of AD

Based on these findings in mice with genetic ATM deficiency, we next tested mouse models of Alzheimer’s disease to determine whether our hypothesis for a reduction of ATM might be present. We tested three different AD transgenic models. R1.40 mice carry a single (full-length) APP transgene; PS/APP mice carry an APP cDNA transgene plus a second presenilin-1 (PSEN1) cDNA transgene; triple-transgenic animals (3xTg) carry APP and PSEN1 cDNA transgenes plus an additional MAPT gene. As R1.40 and PS/APP mice are on the same genetic background, we used their wild-type littermates as controls. Neuronal cell cycle events are found in many different AD models (Li et al., 2011b), which is consistent with our hypothesis. We next asked whether indications of ATM deficiency might also be found.

**Figure 2.**
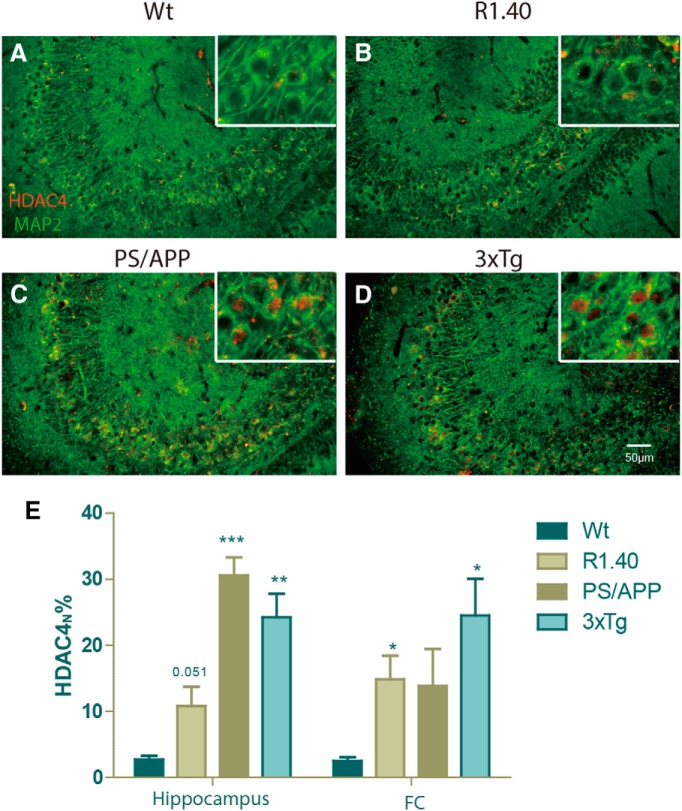
Neuronal ATM level is reduced in mouse models of AD. Sagittal brain sections from three AD mouse models and wild-type controls (age, 13-14 months) were double immunostained for HDAC4 (red) and Map2 (green). Scale bar, 50 μm. ***A–D***, Wild-type mice (Wt; ***A***), R1.40 APP transgenic mouse (***B***), PS/APP double-transgenic mouse (***C***), and 3xTg triple-transgenic mouse (***D***). ***E***, Quantification of the percentage of neurons with HDAC4_N_ found in the frontal cortex and hippocampus of each model examined. Difference determined by *t* test, *p < 0.05, **p < 0.01, ***p < 0.001 (*n* = 3-4).

As would be expected if ATM function were impaired, we found enhanced nuclear HDAC4 (HDAC4_N_) in hippocampal pyramidal neurons of all three models (Fig. 2*B–D*); the background in the wild-type mouse was quite low (Fig. 2*A*). Evidence for reduced ATM also extended to the frontal cortex, and quantification of the percentage of HDAC4_N_ neurons in the two areas (Fig. 2*E*) confirmed that in all three AD models there was clear evidence for a loss of neuronal ATM level. The weakest signal came from the R1.40 mouse, but this is also the transgenic line in which the pathology is slowest to develop (Lamb et al., 1999). Together, these data from the mouse support the hypothesis that neurons subjected to the chemistry of the AD brain display phenotypes that suggest loss of ATM function.

### Nuclear accumulation of HDAC4 found in human AD

**Figure 3.**
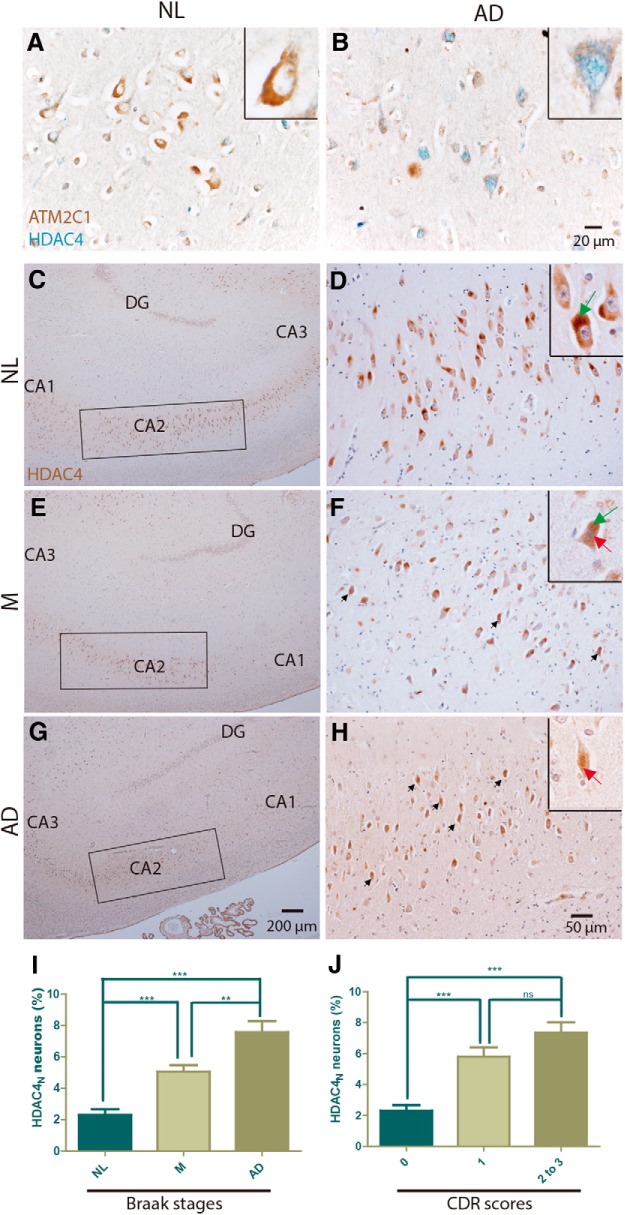
HDAC4_N_ increases with Braak stage in CA2 hippocampal pyramidal cells. ***A***, ***B***, Representative paraffin sections double labeled with ATM 2C1 (brown) and HDAC4 (blue). Signals of ATM 2C1 were strong in neurons of NL (***A***) but not of AD (***B***) frontal cortex. HDAC4 was found predominantly in cytoplasm in NL brains, but took up a nuclear location in AD brains in neurons with weak ATM 2C1 signal. Scale bar, 20 μm. ***C–H***, Paraffin sections of human hippocampus were immunostained with HDAC4 (brown) and counterstained with hematoxylin (blue/purple). ***C***, ***E***, ***G***, Low-magnification images showing the frequency of HDAC4 in the CA2 subfield (marked by rectangles). Scale bar, 200 μm. ***D***, ***F***, ***H***, Higher-magnification images showing cells with the HDAC4_N_ phenotype (arrows). Scale bar, 50 μm. Different Braak stages are represented. ***C*** and ***D*** are from NL case patients (Braak stages 0-2); ***E*** and ***F*** are from M subjects (Braak stages 3-4); and ***G*** and ***H*** are from AD subjects (Braak stages 5-6). Insets in the top right corner of ***D***, ***F***, and ***H*** offer enlargements of representative neurons showing the different phenotypes. Green arrows, HDAC4_C_; red arrows, HDAC4_N_. ***G***, HDAC4_N_ percentage in CA2 neurons as a function of Braak stages. Difference determined by unpaired *t* test: *p < 0.05; **p < 0.01; ***p < 0.001 (*n* = 8-9). ***H***, HDAC4_N_ percentage as a function of CDR scores. CDR0, *n* = 9; CDR1, *n* = 3; CDR2 to 3, *n* = 10. Difference determined by unpaired *t* test, ns > 0.05; ***p < 0.001.

To extend these results to human AD, we examined 27 well characterized autopsy case patients (Table 1). We double immunostained sections from midfrontal gyrus (FC; Brodmann area 8/9) with HDAC4 and ATM antisera. The ATM antibody used was 2C1(A1), which recognizes a stretch of amino acids in the vicinity of the ATM kinase domain (amino acids 2577-3056). In individuals without clinical or pathological signs of AD (Fig. 3*A*), a strong neuronal 2C1 signal was found, almost exclusively in cytoplasm. HDAC4 staining in the same population was weak, and also predominantly cytoplasmic. The situation in the AD brain was substantially different (Fig. 3*B*). The strength of the ATM signal dropped dramatically in some cells, and in these we found that HDAC4_N_ was increased. The HDAC_N_ effect was most prominent in cells with the lowest levels of ATM staining.

We next turned to hippocampus, where the pyramidal neurons of the CA field had the same pattern of staining; HDAC4_N_ was found almost exclusively in cells with reduced ATM (data not shown). We examined the following three Alzheimer’s disease stages: case patients with little or no evidence of disease as well as others who died with mild or advanced AD. At all stages, we found significant levels of HDAC4 immunoreactivity in hippocampal pyramidal cells (Fig. 3*C*,*E*,*G*). In most cells, this staining was located in the neuronal cytoplasm (Fig. 3*D*,*F*,*H*, green arrows, insets). In some neurons, however, as seen in the insets in Figure 3, *F* and *H* (red arrows), HDAC4 was found in the nucleus. In contrast to the wide distribution of neurons with cytoplasmic HDAC4 (HDAC4_C_), most HDAC4_N_ neurons were located in the CA2 subfield (Fig. 3*E–H*). The CA3 and CA4 subfields contained a small number of HDAC4_N_ neurons; very few were seen in CA1. Importantly, the percentage of HDAC4_N_ neurons increased significantly in M individuals with intermediate Braak scores (stages III–IV, *p* < 0.001 compared with stages I–II [NL]) and was higher still in individuals with AD with advanced Braak scores (stages V–VI, *p* < 0.01 compared with M, and *p* < 0.001 compared with NL; Fig. 3*I*).

Although clinical severity is closely correlated with Braak neuropathology, we nonetheless retabulated our results based on the most recently available clinical dementia rating (CDR) score for the subjects examined (Fig. 3*J*). The results were qualitatively similar, although the variance of the scores was larger when the CDR was used as the discriminator. Together, these data are strong evidence that the nuclear translocation of HDAC4 in hippocampal pyramidal cells is an early event in the onset of dementia of the Alzheimer’s type.

### Regional differences in the extent of nuclear accumulation of HDAC4 in AD

Alzheimer’s disease affects neuronal populations in addition to those in the hippocampal formation. These include cells in entorhinal and frontal cortex (Coyle et al., 1983; DeKosky and Scheff, 1990; Arnold et al., 1991; Schröder et al., 1991), as well as the large melanin-containing neurons of the locus ceruleus (Zweig et al., 1988, 1989). The robust association of disease with decreased ATM in hippocampal pyramidal cells led us to ask whether these other brain regions showed a similar correlation. Neurons in the entorhinal cortex proved impossible to analyze as they showed a very low baseline level of total HDAC4 immunoreactivity. Therefore, we turned to material from the LC and the FC collected from the same individuals used for the hippocampal study. Cerebellar cortex was also examined. In each area we quantified, the number of HDAC4_N_ neurons increased with disease stage, along with a decrease in the overall neuronal density (data not shown).

#### Frontal cortex

**Figure 4.**
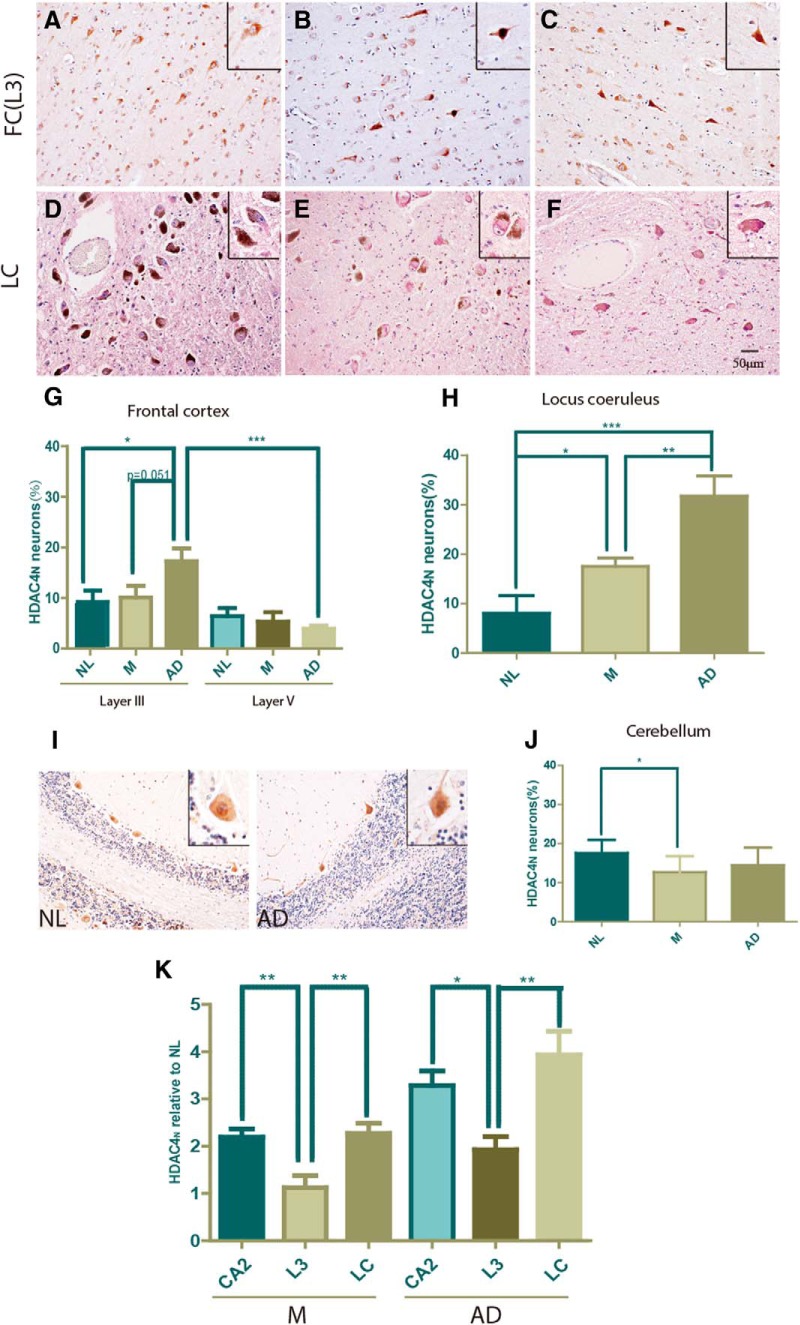
HDAC4_N_ tracks disease severity in multiple brain regions. ***A–C***, Layer III of frontal cortex stained for HDAC4. ***D–F***, Locus ceruleus immunostained for HDAC4 (purple) also shows endogenous melanin (brown). Insets in the top right corner of ***A–F*** are enlargements of representative neurons showing the features scored. ***G***, Abbreviations are the same as in ***A–F***. ***H***, The percentage of total neurons in layers III and V that are HDAC4_N_ ranked by disease stage. The *p* value for differences indicated was assessed by Student’s *t* test, *p < 0.05, **p < 0.01, ***p < 0.001 (*n* = 8-9). ***I***, Cerebellar Purkinje cells were stained by HDAC4 (brown) in NL and AD case patients. Insets represent enlargements of single Purkinje cells with HDAC4_N_. ***J***, Percentage of HDAC4_N_ demonstrates no difference between the cerebellum of NL and AD individuals, but HDAC4_N_ tends to be less frequent in cerebellum of M individuals. Difference determined by *t* test, *n* = 8-9. *p < 0.05. Scale bar, 50 μm. ***K***, Percentages of change of HDAC4_N_ compared with the NL group were calculated for the following three brain regions: the CA2 region of hippocampus (CA2), L3, and LC. Difference determined by *t* test, *p < 0.05, **p < 0.01, ***p < 0.001 (*n* =8-9).

Unlike the entorhinal cortex, total HDAC4 staining was abundant throughout frontal cortex. Indeed, in our elderly subjects, the baseline levels of HDAC4_N_ were several-fold higher (Fig. 4*A–C*), particularly in L3. Compared with NL individuals, however, we found a significant increase in HDAC4_N_ neurons in layer III of individuals with AD (Fig. 4*G*). This situation is reminiscent of the results in the mouse described above, where measurable background levels of HDAC4_N_ were found in wild-type animals even while they were further elevated in *Atm^−/−^* animals (Fig. 1*H*). In human FC, the effect was restricted to layer III; other cortical layers (e.g., layer V) showed little change. This suggests that in AD frontal cortex the loss of ATM occurs late in the disease process, and is consistent with the absence of substantial neuronal cell death in this region at early disease stages. Indeed, tau pathology does not develop in frontal cortex until late Braak stages.

#### Locus ceruleus

LC neurons are easily identified based on their location in the dorsal brainstem, their large size, and the presence of melanin pigment in their cytoplasm (Fig. 4*D–F*). Consistent with the suggestion that the LC is an early target of AD pathogenesis (Braak and Del Tredici, 2011), we found a dramatic stage-specific progression in the percentage of LC neurons that was HDAC4_N_ (Fig. 4*H*). At baseline in NL subjects, HDAC4_N_ neurons accounted for 2-3% of the total, but in Braak stage III–IV disease, the percentage jumped nearly fivefold (*p* < 0.01). In Braak stage V–VI brains, the percentage doubled again (*p* < 0.001) such that fully one-third of the remaining LC neurons were HDAC4_N_. Thus, as in the CA fields of hippocampus, the HDAC4_N_ percentage increased in a tight relation with disease stage, but the effect in the LC is considerably more dramatic. In individuals with AD, the percentage of HDAC4_N_ in LC (31.8 ± 4.0%; *n* = 8) was three times that in the CA2 subfield of the same individuals (7.6 ± 0.7%; *n* = 9). This was so even though the two regions were nearly comparable in the subjects with low Braak scores. These findings underscore the tremendous vulnerability of the LC to the changes that take place during the pathogenesis of AD and extend to a third brain region the correlation between neurodegeneration and evidence for a loss of ATM function.

#### Cerebellum

The cerebellar cortex is a region of the brain that is largely spared in AD. Diffuse plaques appear (Joachim et al., 1989; Yamaguchi et al., 1989), but there is little or no tau pathology and no apparent cell loss (Wegiel et al., 1999; Wolf et al., 1999). To determine whether there might be hidden vulnerability in the form of ATM deficiency, we immunostained cerebellar sections from the same 27 subjects for HDAC4 protein (Fig. 4*I*). We found an unexpectedly high background of HDAC4_N_ in the Purkinje cells of individuals with low Braak scores (NL subjects), nearly twice the percentage of HDAC4_N_ of the layer III neurons from the same subjects (Fig. 4, compare *J*, *G*). The reasons for this background are unknown; however, advancing disease led to little change in its extent. Indeed, if there were any change, it was in the downward direction. Thus, in regions of the AD brain where cell death is less prominent, evidence for a decrease in ATM function is lacking, suggesting a specificity to the effect and correlating with the regional variation found during the progression of AD.

To compare the changes in HDAC4 localization across the different brain regions, we normalized the M and AD case patients to the NL values for that region. The results (Fig. 4*K*) emphasize the early and dramatic rise in the impact of AD on the LC and area CA2 of hippocampus, when viewed from the perspective of ATM loss. We also found that, with only a few exceptions, the same trend of HDAC4_N_ involvement was found in each individual. In hippocampus, the distribution of points was tight in each group, rising with disease stage. In most NL samples, all three regions showed low HDAC4_N_ fractions, with a slight increase already apparent in the LC. For both M and AD case patients, however, the pattern in most individuals was CA2 < L3 < LC.

### ATM protein and message are significantly reduced in AD patients

**Figure 5.**
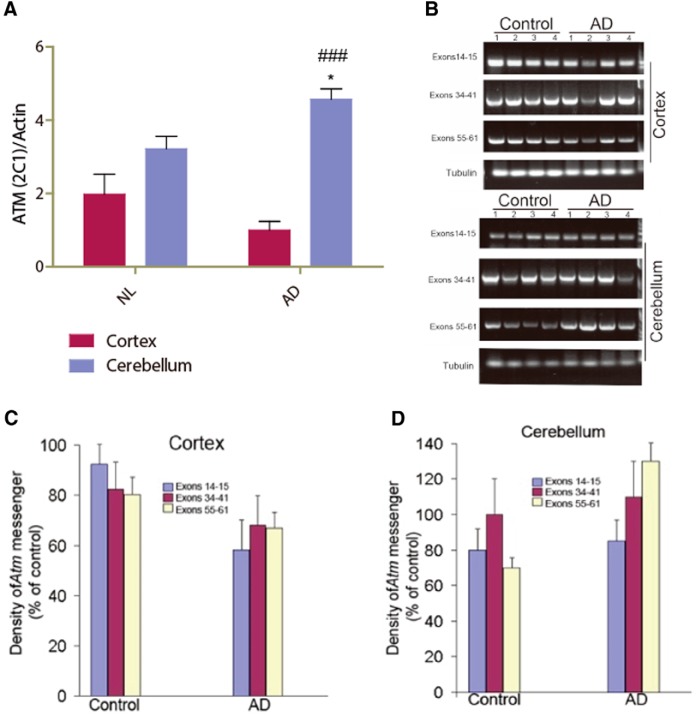
ATM protein and mRNA are reduced in the frontal cortex of AD subjects. Frozen lysates of human frontal cortex and cerebellum from the ADRC at Washington University in St. Louis were analyzed for ATM protein level by Western blot and mRNA level by quantitative PCR. Four subjects were included in each group (control or AD). Subjects less than or equal to Braak stage II were considered as control subjects. ***A***, Quantification of ATM protein level normalized to actin level. Difference between groups for the same region was determined by *t* test, *p < 0.05; difference between regions for the same group, ###*p* < 0.001 (*n* =3-4). ***B***, ATM mRNA level in human brain visualized by RT-PCR. The levels of tubulin message served as a control. ***C***, Comparison of mRNA level in frontal cortex of AD vs control; values are normalized to tubulin (*n* = 4). ***D***, Comparison of mRNA level in cerebellum of AD vs control; values are normalized to tubulin (*n* = 4).

To determine whether the loss of ATM in select cells of the AD brain was the reflection of a more global loss of ATM protein, we used lysates of human frontal cortex and cerebellum to measure ATM protein and mRNA. Western blots were probed with 2C1(A1) antibody. Compared with NL subjects, we found that the level of ATM protein in individuals with AD was lower in the FC (Fig. 5*A*) but higher in the cerebellum (Fig. 5*B*). The decrease in cortex was also seen at the level of ATM message, as determined by RT-PCR with primers that amplify the sequences spanning exons in three different regions of the ATM mRNA. Analysis by RT-PCR showed less ATM message in AD FC (Fig. 5*C*) compared with that in individuals with Braak stage I/II disease. Curiously, the change in cerebellar message was distinctly different depending on which region of the message was assayed (Fig. 5*D*). As with the protein data, however (Fig. 5*B*), and consistent with the HDAC4_N_ percentage, the trend was toward increased ATM presence in cerebellum as AD progresses. For frontal cortex, these data validate the immunocytochemistry (Fig. 3*A*,*B*) and suggest that the HDAC4_N_ findings are part of a larger picture of decreased ATM during the advance of AD that is regionally variable.

### Correlation of multiple indices of reduced ATM level

#### Histone methylation

**Figure 6.**
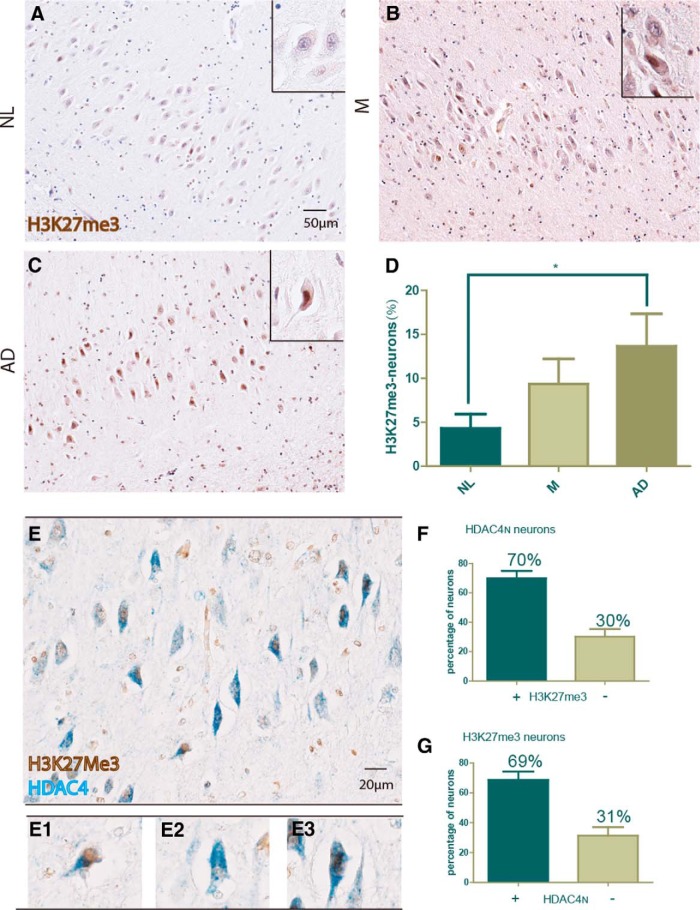
Hippocampal level of H3K27me3 and its relationship to HDAC4_N_. The same set of case patients grouped by Braak stage stained with H3K27me3. ***A–C***, Representative pictures from the CA2 subfield of NL (***A***), M (***B***), and AD (***C***) case patients. Scale bar, 50 μm. ***D***, Quantification of the data from ***A–C*** showing the neurons with an H3K27me3-positive signal as a percentage of total pyramidal neurons. Difference was determined by unpaired *t* test, *p < 0.05 (*n* = 8-9). ***E***, Section from an AD brain double immunostained with H3K27me3 (brown) and HDAC4 (blue). Scale bar, 20 μm. ***E1–E3***, The following different phenotypes of staining scored: H3K27me3-positive, HDAC4_C_ (***E1***); HDAC4_N_ only (***E2***); H3K27me3 plus HDAC4_N_ (***E3***). ***F***, ***G***, Quantification of HDAC4_N_/H3K27me3 double labeling. ***F***, Considering the entire population of HDAC4_N_ neurons, the histogram shows the percentage that were H3K27me3 positive and negative that were scored. ***G***, Considering only the population of H3K27me3-positive neurons, the histogram shows the percentage that was HDAC4_N_ and HDAC4_C_.

The data thus far rely solely on the appearance of HDAC4_N_ (Li et al., 2012) to suggest that the levels of ATM function are reduced along with the levels of protein. We therefore sought a second independent way of demonstrating the loss of ATM from at-risk neurons during the course of AD. EZH2 is a histone methyltransferase that adds three methyl groups to lysine 27 of histone H3 (H3K27me3). Phosphorylation of EZH2 by ATM marks it for degradation, thus keeping its levels low in normal cells (Li et al., 2013). In the absence of ATM, however, nonphosphorylated EZH2 accumulates and the levels of H3K27me3 increase (Li et al., 2013). We immunostained adjacent hippocampal sections from the case patients shown in Figure 3 for H3K27me3 (Fig. 6*A–C*). As with HDAC4_N_ staining, the most prominent H3K27me3 was found in the pyramidal cells of the CA2 region, and its levels were well correlated with the Braak stage pathology (Fig. 6*D*). Thus, a second, unrelated measure leads to the same conclusion: in vulnerable neuronal populations of the AD brain, ATM level is reduced such that the normal epigenetic landscape is changed; HDAC4 is ectopically located in the nucleus (where it deacetylates histone H3 and H4; Li et al., 2012) and H3K27me3 levels are abnormally high.

The appearance of H3K27me3 and HDAC4_N_ were both relatively rare events. Even in AD brains, <10% of the CA2 neurons were HDAC4_N_ and <15% were immunopositive for H3K27me3. Since we presume that both events are caused by a reduction in ATM level, it follows that the two events should be occurring in the same cell. To test this, we double immunostained cells from AD hippocampus for both HDAC4 and H3K27me3 (Fig. 6*E–E3*). The results were clear: of the total number of HDAC4_N_ cells, over two-thirds were also H3K27me3 positive (Fig. 6*F–G*). The reverse was also true: of the total number of H3K27me3-positive neurons, over two-thirds were also HDAC4_N_. Given the relatively rare occurrence of either marker, the odds of both appearing in the same cell by chance are small (1-2%). Thus, two independent markers point to a subpopulation of cells in the AD brain that have a serious deficiency of ATM function.

#### Neuronal cell cycle markers as indices of cell stress

**Figure 7.**
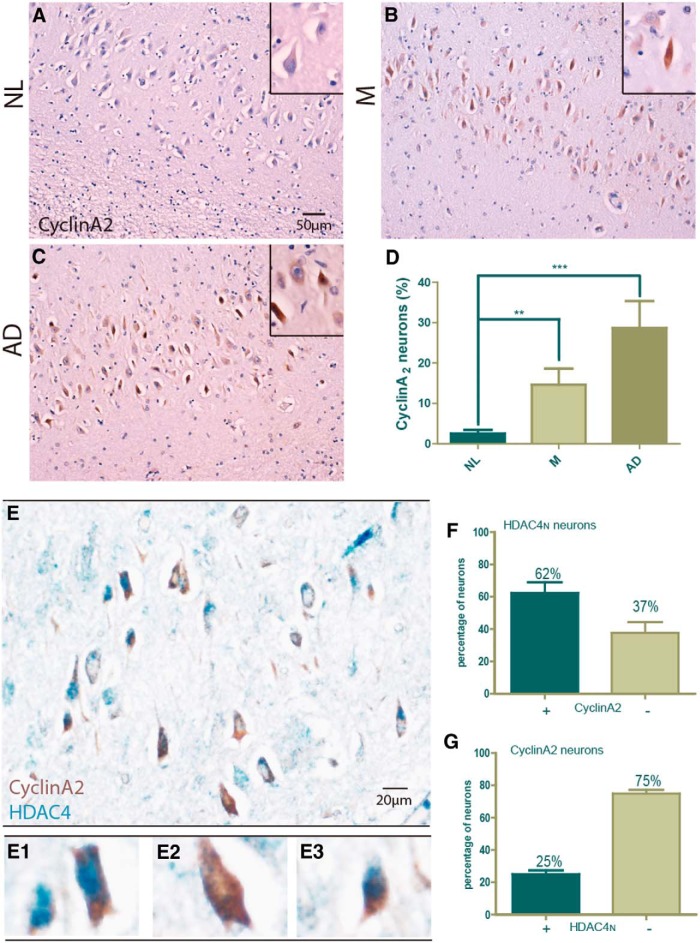
The correlation of cell cycle marker with HDAC4_N_. The same set of case patients grouped by Braak stage stained with cyclin A2 (brown) as a representative marker of ectopic neuronal cell cycle activity. ***A–C***, Representative pictures from the CA2 subfield of NL (***A***), M (***B***), and AD (***C***) case patients. Note that most cyclin A2 was located in the cytoplasm. Scale bar, 50 μm. ***D***, Quantification of the data from ***A–C*** showing the neurons with an H3K27me3-positive signal as a percentage of the total number of pyramidal neurons. Difference determined by unpaired *t* test, *p < 0.05, **p < 0.01, ***p < 0.001 (*n* = 8-9). ***E***, Section from an AD brain double immunostained with cyclin A2 (brown) and HDAC4 (blue). Scale bar, 20 μm. ***E1–E3***, The different phenotypes of staining that were scored: the neuron on the left is HDAC4_N_ only, while the one on the right is both HDAC4_N_ and cyclin A2 positive (in cytoplasm; ***E1***); cyclin A2 only (***E2***); and cyclin A2 plus HDAC4_N_ (***E3***). ***F***, ***G***, Quantification of HDAC4_N_/cyclin A2 double labeling. ***F***, Considering only the population of HDAC4_N_ neurons, the percentage that were cyclin A2 positive and negative were scored. ***G***, Considering only the population of cyclin A2-positive neurons, the histogram represents the percentage that were HDAC4_N_ and HDAC4_C_.

A third neuronal phenotype that is found as a consequence of reduced ATM level is the appearance of ectopic cell cycle events (Yang and Herrup, 2005; Yang et al., 2014). Although this marker has been extensively studied in AD brain, we wished to know whether it too occurred in the cells marked by HDAC4 and H3K27me3. We immunostained our NL (Fig. 7*A*), M (Fig. 7*B*), and AD (Fig. 7*C*) case patients for the cell cycle protein marker cyclin A. As reported previously (Busser et al., 1998; Yang et al., 2006), this marker is elevated in a disease-specific manner during the progression of AD and, like other cyclins, can appear in either the nucleus or, more infrequently, the cytoplasm. Using this marker, we confirmed that cell cycle events were present in the hippocampus, especially in the CA2 subfield, of individuals with AD (Fig. 7*C*). In NL brains, we found low levels of neuronal cyclin A immunostaining; and, as reported earlier (Yang et al., 2003), early stages of dementia (Braak stage III/IV disease) already show elevated neuronal cell cycle activity (cyclin A staining). We performed separate counts of neurons that were stained with cyclin A in the nucleus. This analysis revealed the same trend as total cyclin A (Fig. 7*D*). Double immunostaining with HDAC4 and cyclin A (Fig. 7*E*) revealed once again that nearly two-thirds of the cells that were HDAC4_N_ were also positive for cyclin A (Fig. 7*F*); lower than a 5% overlap would be expected based on chance alone. Thus, a third independent marker of reduced ATM level is found in the same subpopulation of neurons. The combined evidence strongly points to a significant loss of ATM level during the progression of Alzheimer’s disease. One curious additional feature of these findings deserves note. Although the number of cyclin A-positive neurons was tightly correlated with disease stage, we found that of the total number of cyclin A-positive neurons, only 25% were also positive for HDAC4_N_ (Fig. 7*G*). This correlation is still well above chance, but quite different from the strong reciprocal relationship seen with HDAC4_N_ and H3K27me3.

#### No correlation between loss of ATM and phospho-tau

**Figure 8.**
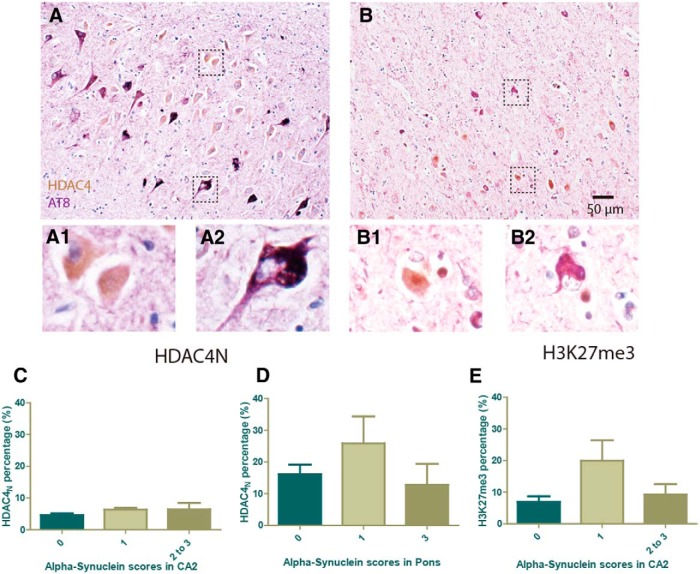
Correlation with other neurodegenerative markers. ***A***, ***B***, Correlation of HDAC4_N_ with tau pathology. ***A***, Representative image of HDAC4 and AT8 (phospho-tau) double labeling in the hippocampus of an AD subject. Insets illustrate the various phenotypes observed, as follows: HDAC4_N_-positive neuron (***A1***); and AT8-positive neuron (***A2***). ***B***, Representative image from the frontal cortex of an AD subject. Insets illustrate the different staining patterns observed, as follows: HDAC4_N_-positive neuron (***B1***); and AT8-positive neuron (***B2***). Scale bar, 50 μm. ***C–E***, Correlation of HDAC4_N_ and H3K27me3 with α-synuclein pathology. Subjects were scored on a semi-quantitative scale for their involvement of α-synuclein pathology. Little correlation was found against this metric of disease severity. ***C***, Percentage of HDAC4_N_ neurons in the CA2 subfield. ***D***, Percentage of HDAC4_N_ (in locus ceruleus) as a function of α-synuclein scores in pons. ***E***, Percentage of H3K27me3 neurons in the CA2 subfield as a function of α-synuclein scores.

ATM loss in multiple brain regions is tightly correlated with the neuropathologically diagnosed Braak stage, which relies heavily on the distribution of tau pathology (Braak and Braak, 1991). We therefore double labeled sections with HDAC4 and tau immunostaining to determine whether the neurons with loss of ATM also showed signs of tau pathology. We were surprised to find no overlap between intracellular phospho-tau and HDAC4_N_ (or any other ATM marker) in either hippocampus (Fig. 8*A–A2*) or in frontal cortex (Fig. 8*B–B2*). Hyperphosphorylated tau was monitored by either PHF1 or AT8, and, while many neurons in CA1, subiculum, entorhinal cortex, and the deeper layers of FC showed robust tau pathology, few of these neurons were HDAC4_N_. We found it noteworthy that in regions such as the CA2 subfield of hippocampus and layer III of FC, where the density of HDAC4_N_ was the highest, the hyperphosphorylated tau signal was weak.

We also scored the same case patients for α-synuclein and Lewy body pathology. Such deposits are abundant in case patients with Parkinson’s disease and dementia with Lewy bodies, but are occasionally found in case patients with AD as well. As with the ATM markers we used, α-synuclein immunoreactivity was high in the CA2 region in the same set of case patients. However, when we expressed either HDAC4_N_ or H3K27me3 as a function of increasing α-synuclein involvement (Fig. 8*C–E*), we found no significant relationship.

## Discussion

Ataxia-telangiectasia is caused by the genetic deficiency of ATM, and results in childhood disability and early death. Most Alzheimer’s disease is sporadic, cannot be ascribed to the malfunction of a single gene, and is rarely detected before the age of 65 years. Although the two diseases strike opposite ends of the human life span, the results presented here suggest that they are related through their patterns of neuronal cell loss. In both conditions, neuronal cell death is highly correlated with a loss of ATM function on a cell-by-cell basis. In the brains of individuals with Alzheimer’s disease, we have used the following four independent methods to document low ATM function: reduction in ATM immunostaining intensity; nuclear translocation of HDAC4; elevation of trimethylation on lysine 27 of histone H3 (H3K27me3); and the appearance of cell cycle proteins, specifically cyclin A2. We find that all four measures support the conclusion that neurons at risk for death in AD undergo a loss of ATM signaling. The high degree of overlap among the markers (Figs. 5, 6) implies that in any one cell, when it occurs, the failure of ATM signaling affects many, if not most, of its cellular functions. Based on these data, we propose that a loss of ATM signaling is a key part of the neurodegeneration mechanism during AD pathogenesis.

The experimental groundwork for this suggestion comes from both *in vitro* and *in vivo* observations of mouse neurons subjected to the partial ATM reduction achieved by *Atm* heterozygosity. Cultured *Atm^+/−^* cortical neurons as well as neurons in the brains of *Atm^+/−^* mice demonstrate cellular abnormalities similar to those found in the AD brain: increased HDAC4_N_, and increased cell cycle activity. A second experimental basis for the A-T/AD linkage comes from our findings in three different AD mouse models. In all three models, we found evidence for reduced ATM function. This is of more than passing significance, as many neuronal phenotypes (e.g., neurofibrillary tangles and neuronal cell death) are not captured in the existing Alzheimer’s disease models. The cellular phenotypes suggesting that ATM deficiency occurs in the AD models provides support for the concept that the altered chemistry of the AD brain produces, as one of its early consequences, a regionally variable loss of ATM function. The precise molecular linkage between the Alzheimer’s abnormalities and the loss of ATM remains unknown.

In neurons of the human AD brain, we find that the loss of ATM is highly correlated with neuronal cell death on a cell-by-cell basis. Indeed, the high degree of overlap among the markers (Figs. 5, 6) implies that in any one cell, when it occurs, the failure of ATM is felt across all domains of its function. The overlap in the markers also supports the conclusion that the changes that we see are not random events in unrelated groups of scattered cells, but rather concerted failures of ATM signaling in a subset of cells. That said, the implications of the one-third of the cell population in which the overlap of the markers is not found is worth considering. The explanation for the single-labeled cells is no doubt partly technical as immunocytochemistry is an imperfect technique. While this explanation may apply to some cells, the reciprocal nature of the incomplete overlap suggests that ATM is at the headwaters of numerous cellular processes, all of which are needed for full neuronal health. Thus, rather than serving as the first step in a single linear pathway, ATM is more like the main gas line to a four-burner gas stove top. As the gas supply (ATM level) diminishes, which of the burners goes out first cannot be predicted; but when the first one goes out, a second will likely follow soon. The value in this analogy is that it emphasizes that while each burner is independent, it is fed by a single source of fuel. Hints that this model is correct can be found in the lack of overlap between the genes identified by Li et al. (2012, 2013) in their ChIPseq analyses of genes whose histone acetylation code is changed after HDAC4 nuclear localization compared with those whose histone methylation code is changed after EZH2 stabilization. Both are ATM-dependent events, yet the ensemble of genes affected are substantially different, two burners fed by the same gas line.

From the perspective of AD neuropathology, it is unexpected that the CA2 subfield of the hippocampus would demonstrate the most dramatic loss of ATM, as this area is not normally viewed as a focus of AD pathogenesis. CA2 neurons have a distinct biochemical identity (Ochiishi et al., 1999; Young et al., 2006; Hanseeuw et al., 2011), and recent evidence suggests a role in synaptic function (Caruana et al., 2012) and social memory (Hitti and Siegelbaum, 2014; Mankin et al., 2015). Intriguingly, it has been reported (Chevaleyre and Siegelbaum, 2010) that the neurons in this region are connected in a reciprocal relationship with CA1 distinct from that with CA3. The suggestion from this relationship is that the CA2 subfield may be the first to experience ATM deficiency, but it then exerts an indirect effect on the other CA subfields, contributing to the pathogenesis of AD.

Our study compared four distinct brain regions in every subject. This more holistic approach reveals several important features of ATM-induced neuronal malfunction. The tight correlation between evidence of ATM reduction and disease state supports the proposition that this reduction is an important feature of the neurodegeneration in AD. The additional finding that the locus ceruleus is heavily involved is significant in several respects. This brainstem nucleus is heavily damaged during the course of AD (Zweig et al., 1989), and it is affected in different mouse models of AD using cell cycle events as a biomarker for neuronal distress (Li et al., 2011b). Based on the appearance of hyperphosphorylated isoforms of tau, it has been speculated (Braak and Del Tredici, 2011) that the damage here begins early. The current study shows that during AD progression the increase in HDAC4_N_ in this region is substantial. The LC of the individuals with little or no AD pathology in our study also had very low levels of HDAC_N_ (2-3%). This observation could simply mean that the events leading to abnormal tau phosphorylation (the basis of the Braak score) precede those that lead to reduced ATM function, but it is also consistent with the conclusion that the earliest tau-related changes mark a parallel cell death pathway of the disease process. This latter alternative fits well with the observed lack of overlap between the level tau pathology (AT8 staining) and the HDAC4_N_ phenotype, either in hippocampus or in frontal cortex (Fig. 6). We did not quantify this relationship in all case patients, but in those case patients we examined it seemed that only a small minority of the cells with tau pathology also were HDAC4_N_. Also of interest is the finding that the loss of ATM function does not correlate with the degree of α-synuclein involvement, a neuropathology most commonly associated with Parkinson’s disease, but often found in AD brains as well. The HDAC4_N_ and H3K27me3 phenotypes were largely unrelated to the extent of the α-synuclein deposits. The suggestion of this finding is that the loss of ATM signaling may be an AD-specific phenomenon.

At first, the idea that the protein responsible for causing A-T is also involved in AD pathogenesis seems improbable. The two diseases themselves could not be more different. AD is a common late-onset dementia that primarily affects the neurons of the neocortex and archicortex, along with several subcortical structures. A-T is a rare early childhood movement disorder that primarily targets the neurons of the cerebellar cortex. Yet the fact that a genetic deficiency of ATM significantly shortens life span means that any individual with A-T will never get old enough to show even the first symptoms of AD. This recalls the evolutionary concept of “antagonistic pleiotropy” (Williams, 1957), a hypothesis proposed to explain the failure of evolution to select against the process of aging (senescence). The idea is that any genetic change that produces “a greater advantage in increasing youthful vigor [even] at the price of vigor later on” cannot be selected against and indeed would likely be selected for. This means that any gene or genes that led to a reduction of ATM late in life (Fig. 5) would be selected for so long as it contributed to “vigor” in the early years of life. This selection would occur even though the same gene might lead to Alzheimer’s later in life.

The suggestion that emerges from this new perspective is that enhancing the level of ATM, especially in brain, would be worth exploring as a novel therapeutic approach to AD. Although boosting enzyme function is normally a more difficult strategy, agents such as chloroquine have been found to increase ATM levels (Schneider et al., 2006) without the induction of DNA damage. Chloroquine has been tested in traumatic brain injury (Cui et al., 2015) and in AD models (Deng et al., 2016). Such studies tend to attribute the effects of the drug to its impact on autophagy and lysosomal function. Yet it would be difficult to exclude that the effect was more directly linked to ATM. Together, our findings represent a fresh and unexpected view into the cell biological mechanisms that contribute to the neurodegenerative events of AD, and offer new and potentially valuable inroads into disease prevention or therapy.

## Synthesis

The decision was a result of the Reviewing Editor Julie Andersen and the peer reviewers coming together and discussing their recommendations until a consensus was reached. A fact-based synthesis statement explaining their decision and outlining what is needed to prepare a revision is listed below.

The authors' have clarified that in a previous publication (Li et al., 2011), the AWB mutation used in the current study leads to modest reductions in levels of ATM protein resulting in lower kinase activity in response to etoposide-induced DNA damage. In addition, the authors' themselves have previously published that heterozygous Atm+/tm1Awb mice have cell cycle issues in their cerebellar Purkinje cells (Yang and Therefore the heterozygous animals would not be expected to display WT phenotypes. Importantly, the data in the current manuscript suggests that even small reductions in neuronal ATM function are enough to induce the phenotypes akin to those observed in the frontal cortex of human Alzheimer's disease (AD) patients where mild ATM loss may be a contributing factor. The authors' have agreed to modify the manuscript text to clarify this point.

Findings from these current studies fit well with the stated goals of eNeuro towards publishing studies that are technically solid but which "present new observations that may not yet have mechanistic underpinnings..." Given that this suggests a potentially novel mechanism for AD pathogenesis, publication of this work will likely spark important new discussions in the field.
